# Multi-Frequency Entropy for Quantifying Complex Dynamics and Its Application on EEG Data

**DOI:** 10.3390/e26090728

**Published:** 2024-08-27

**Authors:** Yan Niu, Jie Xiang, Kai Gao, Jinglong Wu, Jie Sun, Bin Wang, Runan Ding, Mingliang Dou, Xin Wen, Xiaohong Cui, Mengni Zhou

**Affiliations:** 1 College of Computer Science and Technology (College of Data Science), Taiyuan University of Technology, Taiyuan 030024, China; niuyan@tyut.edu.cn (Y.N.); xiangjie@tyut.edu.cn (J.X.); k0error@163.com (K.G.); sunjie0086@link.tyut.edu.cn (J.S.); wangbin01@tyut.edu.cn (B.W.); 2023510380@link.tyut.edu.cn (R.D.); doumingliang@tyut.edu.cn (M.D.); cuixiaohong@tyut.edu.cn (X.C.); 2 Research Center for Medical Artificial Intelligence, Shenzhen Institute of Advanced Technology, Chinese Academy of Sciences, Shenzhen 518055, China; wujinglong5@gmail.com; 3 School of Software, Taiyuan University of Technology, Taiyuan 030024, China; cquwx0214@163.com

**Keywords:** multi-frequency entropy, electroencephalography, multivariate entropy, nonlinear time series, complex dynamics

## Abstract

Multivariate entropy algorithms have proven effective in the complexity dynamic analysis of electroencephalography (EEG) signals, with researchers commonly configuring the variables as multi-channel time series. However, the complex quantification of brain dynamics from a multi-frequency perspective has not been extensively explored, despite existing evidence suggesting interactions among brain rhythms at different frequencies. In this study, we proposed a novel algorithm, termed multi-frequency entropy (mFreEn), enhancing the capabilities of existing multivariate entropy algorithms and facilitating the complexity study of interactions among brain rhythms of different frequency bands. Firstly, utilizing simulated data, we evaluated the mFreEn’s sensitivity to various noise signals, frequencies, and amplitudes, investigated the effects of parameters such as the embedding dimension and data length, and analyzed its anti-noise performance. The results indicated that mFreEn demonstrated enhanced sensitivity and reduced parameter dependence compared to traditional multivariate entropy algorithms. Subsequently, the mFreEn algorithm was applied to the analysis of real EEG data. We found that mFreEn exhibited a good diagnostic performance in analyzing resting-state EEG data from various brain disorders. Furthermore, mFreEn showed a good classification performance for EEG activity induced by diverse task stimuli. Consequently, mFreEn provides another important perspective to quantify complex dynamics.

## 1. Introduction

The brain is a highly complex dynamic system. The complexity research in the realm of nonlinear dynamical systems has markedly propelled our understanding of electroencephalographic features [1,2,3,4]. Entropy, which serves as a nonlinear dynamical metric for assessing the complexity of time series, has been extensively applied in diverse fields, including signal analysis, data compression, and feature extraction, among others [5,6,7,8,9]. The measurement of entropy within physiological signals is of significant importance for monitoring the physiological states within a system. Consequently, researchers have developed entropy-based algorithms for the analysis of EEG signals, with the expectation that they will yield more precise and comprehensive insights into the mechanisms of neural activity [10,11].

Entropy algorithms can be divided into two major categories based on their theoretical foundation: one category is algorithms based on Shannon entropy, which directly evaluate the average uncertainty of information; the other category is algorithms based on conditional entropy, which reveal complex dynamic relationships within or between signals by calculating the residual uncertainty under certain conditions [7]. These methods provide us with a way to deeply understand the dynamic characteristics of physiological signals from an information theory perspective. According to the single-channel analysis or multi-channel analysis of the EEG signal, entropy algorithms are further subdivided into univariate entropy and multivariate entropy algorithms.

In univariate entropy algorithms, permutation entropy (PerEn) [12] and sample entropy (SampEn) [13] are two techniques that are widely used. SampEn is defined as the negative natural logarithm of the conditional probability that a signal of a specific length N, after repeating itself within a tolerance r for m samples, will continue to do so for m + 1 samples [13]. PerEn was proposed by Bandt and Pompe [12] and served as a complexity measure based on comparing neighboring values of each point and mapping them to ordinal patterns [12,14]. In recent years, PerEn and SampEn have been widely employed to analyze cerebral activity and elucidate the underlying mechanisms of neurological disorders [15,16,17,18,19].

It is important to note that most physiological and pathological processes, accompanied by a multitude of non-physiological signals, involve complex interactions among multiple discrete processes [20,21]. Accordingly, multivariate PerEn (mvPerEn) [22] and multivariate SampEn (mvSampEn) [23] algorithms were proposed to measure the complexity in non-stationary multivariate signals based on univariate entropy. Multivariate entropy algorithms are advantageous due to their ability to capture inter-variable dependencies, aggregate information, and detect nonlinear interactions, as well as their robustness in analyzing complex systems and multivariate time-series data [23,24,25]. Researchers frequently consider channels as independent variables of multivariate entropy algorithms [24,25,26,27,28].

The complexity of neural activity is intricately associated with the interaction of information within and across various frequency bands [29,30]. Oscillatory activities across various frequency bands play a pivotal role in neuronal regulation, with high-frequency brain activities typically associated with localized cortical processing and low-frequency brain rhythms dynamically influenced by both external sensory inputs and internal cognitive processes across widespread brain areas [29,30]. Prior research has demonstrated that interactions occur between brain rhythms of varying frequencies and diverse signals, with preliminary findings already obtained [31,32,33,34]. Studies found that conventional multivariate entropy methods often fall short of accurately distinguishing between the various sleep stages in diagnosing disorders such as sleep disturbances, and by analyzing the correlations between the theta and delta frequency bands, a more precise identification of deep sleep and REM sleep stages could be achieved [35]. Therefore, the investigation of signals within different and between different frequency bands is of paramount importance. However, no multivariate entropy algorithm has yet been developed specifically for addressing the distinct characteristics of multi-frequency bands.

Building on this, we introduced a multi-frequency perspective and proposed a multi-frequency entropy algorithm (mFreEn). The mFreEn, by combining mvPerEn based on Shannon entropy with mvSampEn based on conditional entropy, preserves temporal information when integrating data from different frequency bands. This approach offers the flexibility to select multiple frequency bands of interest for analysis. To assess the efficacy of this novel algorithm, experiments were conducted using simulated data and real EEG data. The sensitivity and noise robustness of mFreEn were analyzed by simulated data. The diagnostic performance was analyzed by real EEG data. The findings elucidated four advantages of mFreEn: (1) elevated sensitivity, (2) reduced parameter dependency, (3) good noise resistance, and (4) good classification performance.

## 2. Materials and Methods

**Figure 1:**
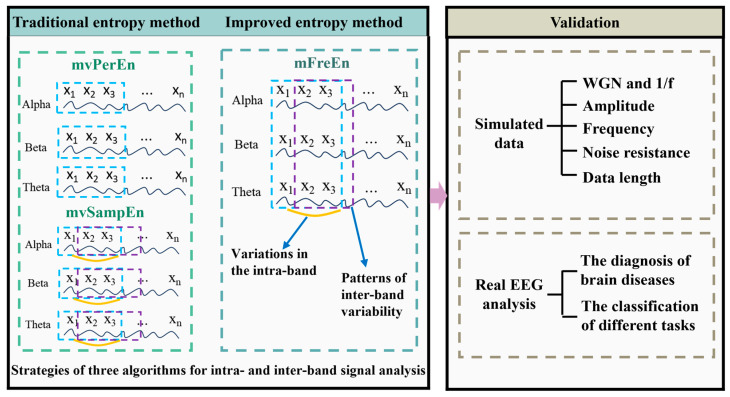
The technical route of this study.

The technical route of this study is depicted in Figure 1. This section firstly introduces the algorithms mvPerEn and mvSampEn, as well as the newly proposed mFreEn algorithm. Second, the simulated data and the real EEG data are presented for the validation of the proposed algorithm.

### 2.1. Multivariate Entropy

#### 2.1.1. Multivariate Permutation Entropy (mvPerEn)

mvPerEn is an extension of PerEn, which is a complexity analysis tool for multivariate time series [22]. For a *g*-variate time series ${U=\{u_{g,l}\}}_{l=1,\;2,\;\ldots ,\;N}^{g=1,\;2,\;\ldots ,\;k}$, by introducing the embedding dimension *m* and time delay *τ*, the multivariate embedded reconstruction is defined as follows:

(1) $$U_{m}\left(i\right)=\left\{u_{g,i},u_{g,i+\tau },\ldots ,u_{g,i+\left(m-1\right)\tau }\right\},\;1\leq i\leq N-(m-1)\tau$$

We uniformly set the embedding dimension at *m* and the delay at *τ* for each variate, then arrange the *m* reconstructed components of **U***_m_* (i) in ascending order:

(2) $$\left[u(i+(j_{1}-1)\tau )\leq u(i+(j_{2}-1)\tau )\leq \cdots \leq u(i+(j_{m}-1)\tau )\right]$$

This gives the arrangement of the vector **U***_i_* as {$j_{1},j_{2},\cdots {,j}_{m}$}, which is one of the *m*! methods of arrangement. $j_{1},j_{2},\cdots {,j}_{m}$ represents an index of the columns of each element in the reconstructed component. The occurrences of each permutation within the entire sequence are counted, and the relative frequencies of these permutations are computed to serve as their probabilities: ${\;p}_{r},\;r=1,\;2,\;\ldots ,m!$.

(3) $${\;p}_{r}=\frac{\#\{i|i\leq N-(m-1)\tau ,type(X_{m}(i))=\pi_{r}\}}{\left(N-(m-1)\tau \right)}$$

The mvPerEn defined by Shannon’s theorem is as follows:

(4) $$H_{p}=-\sum_{r=1}^{m!}p_{r}lnp_{r}$$

When ${\;p}_{r}$ = 1/*m*!, *H_p_*(*m*) reaches the maximum value of ln(*m*!) so that it can be passed through ln(*m*!). *H_p_*(*m*) is standardized, that is,

(5) $$mvPerEn(U,m)=H_{p}/\ln(m!)$$

#### 2.1.2. Multivariate Sample Entropy (mvSampEn)

Ahmed and Mandic presented mvSampEn [23]. It is based on SampEn and is widely used in physical and physiological signals. For a *g*-variate time series${\;U=\{u_{g,l}\}}_{l=1,\;2,\;\ldots ,\;N}^{g=1,\;2,\;\ldots ,\;k}$ of the data length *N*, let **M** = [*m*_1_, *m*_2_, *…*, *m_g_*] ∈ *R^g^* be the embedding vector, and let **τ** = [*τ*_1_, *τ*_2_, …, *τ_p_*] be the time lag vector. The multivariate embedding vector **U***_m_*(*i*) is expressed as follows:

(6) $$\begin{array}{c} U_{m}(i)=[u_{1,i},u_{1,i+\tau_{1}},\ldots ,u_{1,i+(m_{1}-1)\tau_{1}},u_{2,i},u_{2,i+\tau_{2}},\\ \ldots ,u_{2,i+(m_{2}-1)\tau_{2}},u_{g,i},u_{g,i+\tau_{g}},\ldots ,u_{g,i+(m_{g}-1)\tau_{g}}] \end{array}$$

We form (*N* − *n*) composite delay vectors ***U****_m_*(*i*) ∈ *R^m^*, where i = 1, 2, …, *N* − *n*, and *n* = max{**M**} × max{**τ**}. The Chebyshev distance **d***m ij* between the multivariate embedding vector **U***_m_*(*i*) and **U***_m_*(*j*) is calculated using the following formula:

(7) $$d_{ij}^{m}=d[U_{m}(i),U_{m}(j)]=\max_{t=1,\ldots ,m}\{|u(i+t-1)-u(j+t-1)|\},i\neq j$$

Given the similarity tolerance *r*, the probability of **d***_ij_* ≤ *r*, *i* ≠ *j* is $\phi_{i}^{m}=\frac{1}{N-n-1}P_{i}$, and then the global quantity is defined:

(8) $$\varphi^{m}(r)=\frac{1}{N-n}\sum_{i=1}^{N-n}\varphi_{i}^{m}(r)$$

The embedding dimension can be extended from *m* to *m* + 1 in *g* different ways, and this expansion process can be expressed as transforming [*m*_1_, *m*_2_, …, *m_g_*] into [*m*_1_, …, *m_k+_*_1_, …, *m_g_*]. As a result, *g* new multivariate embedding vectors **U***_m_*_+1_(*i*) are obtained. By repeating the above steps, the global quantity with an embedding dimension of *m* + 1 is acquired:

(9) $$\varphi^{m+1}(r)=\frac{1}{g\times (N-n)}\sum_{i=1}^{g\times (N-n)}\varphi_{i}^{m+1}(r)$$

The mvSampEn defined by Shannon’s theorem is as follows:

(10) $$mvSampEn(U,m,\tau ,r)=-\ln(\phi^{m+1}(r)/\phi^{m}(r))$$

#### 2.1.3. Multi-Frequency Entropy (mFreEn)

This section introduces the mFreEn algorithm proposed in this study. The inputs to the mFreEn algorithm are normalized time series of multiple frequency bands, usually obtained by filtering the signal from a lead. As filtering is a pre-processing step prior to data analysis, we do not specify here which filtering method should be used. In this study, the eegfilt function in the EEGLAB toolbox was used to filter the data into the specified frequency bands.

**Figure 2:**
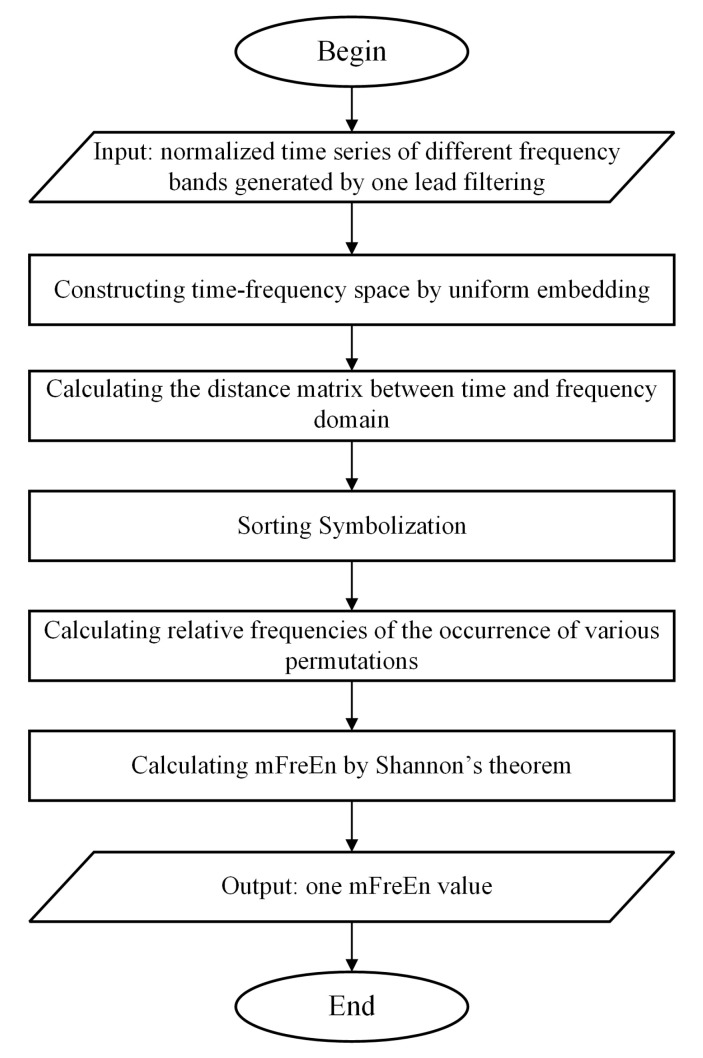
The flow diagram of mFreEn algorithm.

Firstly, we computed amplitude changes between successive time points within each frequency band. Continue, the concept of symbolization was introduced to enumerate the permutation patterns of relative amplitude changes across various frequency bands. The algorithm flowchart of mFreEn is shown in Figure 2. Taking the alpha, beta, and theta frequency bands as examples, we consider multiple frequency bands’ time series **U** with a length of *N*:

(11) $$U=\left[\begin{array}{cccc} \begin{array}{c} u_{A}(1)\\ u_{B}(1)\\ u_{T}(1) \end{array} & \begin{array}{c} u_{A}(2)\\ u_{B}(2)\\ u_{T}(2) \end{array} & \begin{array}{c} \ldots \\ \cdots \\ \cdots \end{array} & \begin{array}{c} u_{A}(N)\\ u_{B}(N)\\ u_{T}(N) \end{array} \end{array}\right]$$

Here, $u_{A}(i),u_{B}(i),\;and\;u_{T}(i)$, respectively, represent each time point corresponding to the alpha, beta, and theta frequency bands.

With a predefined embedding dimension *m* and time delay *τ*, we construct a time-frequency space that includes multiple frequency bands: $Y_{m}(1),Y_{m}(2),\ldots ,Y_{m}(N-m+1)$.

(12) $$Y_{m}(i)=\left[\begin{array}{cccc} \begin{array}{c} u_{A}(i)\\ u_{B}(i)\\ u_{T}(i) \end{array} & \begin{array}{c} u_{A}(i+\tau )\\ u_{B}(i+\tau )\\ u_{T}(i+\tau ) \end{array} & \begin{array}{c} \ldots \\ \cdots \\ \cdots \end{array} & \begin{array}{c} u_{A}(i+(m-1)\tau )\\ u_{B}(i+(m-1)\tau )\\ u_{T}(i+(m-1)\tau ) \end{array} \end{array}\right]$$

Here, 1≤i≤N−(m−1)τ. The maximum absolute difference $d_{ij}^{m}$ between the corresponding frequency bands of the pairwise time-frequency spaces is calculated as follows.

(13) $$d_{ij}^{m}=d[Y_{m}(i),Y_{m}(j)]=\{D_{ij}^{m}(A),\;D_{ij}^{m}(B),D_{ij}^{m}(T)\}\;=\{max\{|u_{A}(i+k)-u_{A}(j+k)|\},max\{|u_{B}(i+k)\;-u_{B}(j+k)|\},max\{|u_{T}(i+k)-u_{T}(j+k)|\}\}$$

Here, *k* = 0, *τ*, 2*τ*, …, (*m* − 1)*τ*. The distance attributes for each frequency band within the aforementioned distance matrix are organized in ascending order based on their magnitude. We match these ordered distances with the *g*! possible patterns *π_h_*, *h* = 1, 2, …, *g*!, where *g* represents the number of frequency bands. The number of occurrences of various permutations in the entire sequence is counted, and the relative frequencies of the occurrence of various permutations are calculated as their probabilities: $p_{t},\;t=1,\;2,\;\ldots ,\;g!$.

(14) $$p_{t}=\frac{\#\{i|i\leq N-(m-1)\tau ,type(U_{m}(i))=\pi_{t}\}}{\left(N-(m-1)\tau \right)}$$

The mFreEn defined by Shannon’s theorem is as follows:

(15) $$H_{p}=-\sum_{t=1}^{\;g!}p_{t}lnp_{t}$$

When $p_{t}=1/g!$, *H_p_*(*m*) attains its maximum value of ln(g!), Therefore, we normalize the entropy value by dividing it by ln(g!):

(16) $$mFreEn(U,m)=H_{p}/\ln(g!)$$

#### 2.1.4. Algorithm Parameter Settings

The study proposed an improved multivariate entropy method, mFreEn, for analyzing the complexity of multiple frequency bands generated by filtering under a single channel and compared it with mvPerEn and mvSampEn. Since the multi-frequency entropy proposed in this study was computed by uniting multiple frequency band signals for multivariate embedding space construction (as shown in Figure 1), uniform embedding was used. For consistent comparisons, uniform embedding was also adopted for both mvPerEn and mvSampEn.

The parameters of the three multivariate entropy algorithms were set as follows, based on relevant references. In mvPerEn, each channel’s time delay *τ* was set to 1 [36,37,38]. In the mvSampEn algorithm, each channel’s *τ* was set to 1, and the similarity tolerance r was set to r = 0.15 × std (standard deviation of the covariance matrix) [39,40,41]. For the mFreEn algorithm, each channel’s *τ* was set to 1. Studies have found that *τ* has little effect on the performance of the algorithm [42,43]. Therefore, in this study, for all three algorithms, *τ* was set to 1. All three algorithms require a parameter setting for the embedding dimension *m*. The value of *m* generally ranges from 2 to 7. The effect of this parameter on the algorithms was investigated in the experimental part of this study using simulated data.

### 2.2. Datasets and Data Preprocessing

#### 2.2.1. Simulated Data Generated Using the MIX Model

The MIX model, which comprises periodic and stochastic noise signals in a specific proportion, was utilized to analyze the effects of different parameters on entropy values [44].

(17) MIX(p)=(1−p)×X+p×Y

(18) $$X(t)=A\sin(2\pi t/f_{S})$$

The MIX signal is obtained by mixing two signals, including the periodic signal X and the random signal Y. The random noise signal Y is a sequence of length *N* uniformly distributed between $-\sqrt{3}$ and $\sqrt{3}$. The MIX model is based on the assumption that the random signal follows a standard normal distribution with mean 0 and variance 1. In a standard normal distribution, approximately 99.7% of the data fall within 3 standard deviations of the mean. Since the standard deviation of the normal distribution is 1, the range of the random signal in the MIX model is between −3 and 3, which corresponds to the values of $-\sqrt{3}$ and $\sqrt{3}$. In the periodic signal X, *N* × *p* points are replaced with the random noise signal Y, where *p* is a probability parameter ranging from 0 to 1. As *p* increases, the complexity of the data generated by the MIX model also increases. The variable X denotes the periodic signal defined by the subsequent formula. The symbols *A* and *f_s_* represent amplitude and frequency, respectively.

#### 2.2.2. Resting-State EEG

This study used two sets of resting-state EEG data for multi-frequency entropy analysis.

(1) Parkinson’s disease dataset

EEG data from 14 Parkinson’s disease (PD) patients (mean age: 70.5 ± 8.7 years; 6 males and 8 females) and 14 control subjects (mean age: 70.5 ± 8.7 years; 6 males and 8 females) were used at the University of Iowa (the data can be obtained from the website https://bit.ly/3pP1pts and accessed on 5 April 2023) [45]. The EEG was captured using sintered Ag/AgCl electrodes within a frequency range of 0.1–100 Hz and a sampling rate of 500 Sa/s, utilizing a 64-channel Brain Vision system. Further information regarding the dataset is available in Supplementary Materials and the referenced literature.

(2) Depressive tendency’s dataset

EEG signals were recorded from twenty-eight healthy volunteers at the Shenzhen Institute of Advanced Technology, Chinese Academy of Sciences. The participants included 14 individuals without depressive tendencies (mean age: 25.21 ± 2.80 years; 4 males and 10 females) and 14 individuals with depressive tendencies (mean age: 27.78 ± 5.14 years; 3 males and 11 females), as assessed by the Beck Depression Inventory Scale scores. The recordings took place in a quiet, dimly lit room. Participants self-reported as right-handed and had either normal vision or corrected-to-normal vision. None of the participants reported a history of neurological problems. Prior to testing, informed consent was obtained from all participants. EEG data were captured using 32 electrodes by the international 10-10 system. Detailed information regarding the dataset is available in Supplementary Materials.

#### 2.2.3. Task-State EEG

The task-state dataset including thirty-two healthy volunteers (mean age: 24.63 ± 3.06 years; age range: 21–32 years; 24 males and 8 females) were recorded from Okayama University in Japan. In the task-state EEG study of visual perception, spatial frequencies (SF) are thought to convey different types of information for visual processing: low SF (LSF) values represent large-scale variations in luminance changes (coarse visual information), whereas high SF (HSF) values represent tighter gradients (fine visual information). The EEG data were digitized at a sampling rate of 500 Sa/s. The main experiment was a within-subject design comprising 4 different conditions: basic SF (BSF), LSF, medium SF (MSF), and HSF. Stimuli were greyscale images of different conditions, scaled to 600 × 600 pixels. Further information regarding the dataset is available in Supplementary Materials.

#### 2.2.4. Data Preprocessing

EEG preprocessing was conducted offline using the EEGLAB v2019.1 toolbox MATLAB R2018b (MathWorks, Natick, MA, USA). Data underwent bandpass filtering from 0.1 to 40 Hz. Apart from the channels previously excluded (specific exclusion details for each dataset can be found in Supplementary Materials), the Independent Component Analysis (ICA) algorithm in the runica function of EEGLAB is used to separate artifacts in EEG signals, removing major artifact components such as eye movements, muscle, and heartbeat.

Additionally, task-related EEG data were segmented from 800 ms pre-stimulus to 1200 ms post-stimulus onset. The reference was recalculated offline using the average of all electrodes. Following re-referencing, trials underwent baseline correction using the prestimulus interval (−200 ms to 0 ms) as the reference period. For both task-state and resting-state data, EEG signals were decomposed into theta, alpha, and beta frequency bands using the short-time Fourier transform (STFT) and bandpass filtering techniques, respectively.

It should be noted that the signal values in different frequency bands need to be normalized for each channel. This is mainly due to the fact that when analyzing the data it is possible that the amplitude of one band is several times that of others, thereby biasing the distribution of permutations.

#### 2.2.5. Design and Analysis of Experiments

(1) Analysis of simulated data

Referring to the studies on multivariate entropy [46,47], we simulated different combinations of signals to verify the performance of the algorithm. Different noise signals as well as MIX signals are widely used as simulated signals.

To evaluate the ability of these algorithms to analyze the complexity of time series with varying frequency distributions, we produced white Gaussian noise (WGN) and 1/f noise. WGN is a random signal with equal energy across all frequencies, but 1/f noise is a signal with a power spectral density inversely proportional to the frequency. The data length was set to 1500, and the generation of the time series was repeated 20 times. We filtered the simulated signals into theta (4–8 Hz), alpha (8–13 Hz), and beta (13–30 Hz) frequency bands.

To investigate the discriminative capacity of the three algorithms for sinusoidal signals across different frequency bands with variable amplitudes, we used an MIX model to simulate signals. Firstly, sinusoidal signals with frequencies of 6 Hz, 10 Hz, and 20 Hz were used to simulate the theta, alpha, and beta frequency bands, respectively. Subsequently, three amplitude levels $A=[\sqrt{2},2\sqrt{2},3\sqrt{2}]$ were selected for each frequency band, resulting in a total of six distinct cases. To investigate the effect of different frequencies on entropy values, the frequencies of the beta band were set at 15 Hz, 20 Hz, 25 Hz, and 30 Hz. Concurrently, the theta and alpha bands were consistently set at 6 Hz and 10 Hz, respectively.

To assess the noise resistance of the three algorithms, white noise (5 dB, 10 dB, 15 dB, 20 dB, and 25 dB) was superimposed at varying signal-to-noise ratios (SNRs) on the MIX model data to generate simulated signals. In addition, we simulated MIX signals of different lengths to explore the effect of data length on the algorithm.

Finally, signals of different frequency bands from various signal combinations were used as input for the mvPerEn, mvSampEn, and mFreEn algorithms to calculate entropy values.

(2) Analysis of real EEG data

A total of three sets of real EEG data were used in this study, two were resting-state EEG datasets and one was a task-state EEG dataset. After the pre-processing steps, signals of different frequency bands of each electrode were used as an input to the multivariate entropy algorithm to calculate the entropy value. Finally, the classification performance of the algorithm was verified by using different classifiers.

## 3. Results

### 3.1. Evaluation of Algorithms Based on Simulated Data

#### 3.1.1. The Sensitivity Analysis for White Gaussian Noise and 1/f Noise

**Figure 3:**
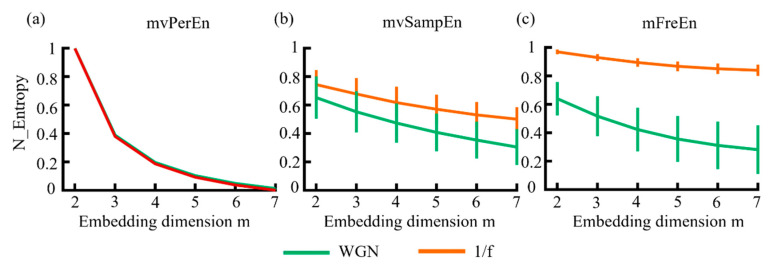
The mean and SD of entropy values were obtained using (**a**) mvPerEn, (**b**) mvSampEn, and (**c**) mFreEn from various WGN and 1/f noise time series with a length of 1500 data points each.

In this study, WGN and 1/f noise were simulated to verify whether the algorithm can distinguish signals with different frequency distributions. To facilitate comparison, the entropy values of algorithms were normalized to [0 1] and the subsequent results were also normalized. The mean and standard deviation (SD) of entropy values were computed. The results are illustrated in Figure 3. As shown in Figure 3, for the three algorithms, the entropy values of 1/f noise and WGN decreased almost monotonically as the embedding dimension increased. Among these, mvPerEn was completely unable to distinguish between WGN and 1/f noise, despite its smaller SD. The mvSampEn could differentiate between the two signals, but the entropy curve exhibited aliasing in all embedding dimensions. As can be seen in Figure 3, mFreEn effectively distinguished between the two signals in all embedding dimensions. Overall, mFreEn demonstrated better performance compared to mvPerEn and mvSampEn in distinguishing signals with different frequency distributions.

#### 3.1.2. Analysis of Amplitude Detection Capability Based on MIX Model

**Figure 4:**
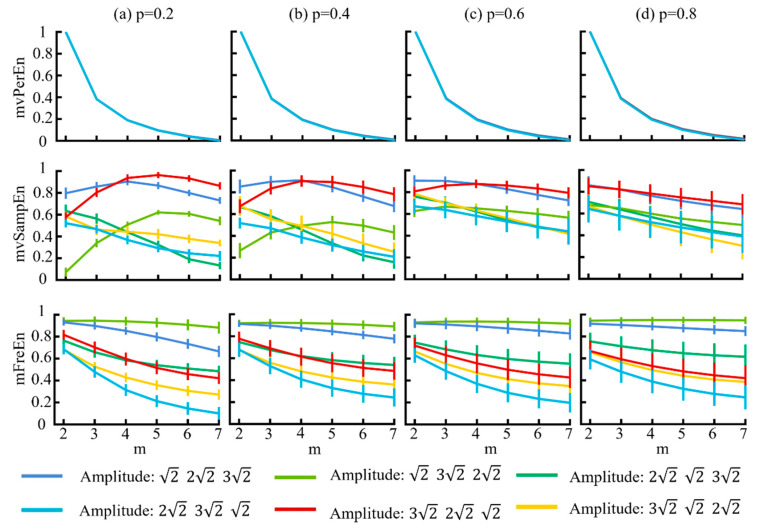
The mean and SD of normalized entropy values of the three algorithms were computed with variable amplitude and a length of 1500 sample points. The results for different *p* values are displayed in panels (**a**–**d**).

Using the MIX model, we generated six different amplitude combinations of signals to verify the sensitivity of the algorithm. The model parameters were delineated as follows: the probability parameter *p* was set as [0.2, 0.4, 0.6, 0.8], the sampling rate *f_s_* = 500 Hz, and *t* = 3 s. The simulated signals were filtered into theta (4–8 Hz), alpha (8–13 Hz), and beta (13–30 Hz) frequency bands, and the entropy values were calculated using the three different algorithms. Figure 4 illustrates the variations in entropy across various embedding dimensions *m* and the probability parameters *p*.

Figure 4 illustrates that for the mvPerEn and mFreEn algorithms, the entropy values of the six signals were either nearly constant or exhibit a gradual decline with increasing embedding dimension. However, the entropy variations in the mvSampEn algorithm were quite erratic. The relative magnitude of the entropy values of the six amplitude levels may not be consistent at different values of *m* or *p*. Compared to mFreEn, mvSampEn exhibited greater overlap in the entropy values in some embedding dimensions, and those of mvPerEn also showed overlap across all embedding dimensions. Furthermore, as the probability parameter *p* increased, the entropy results of mvSampEn became more chaotic with greater overlap. Consequently, mvSampEn and mFreEn significantly outperformed mvPerEn in terms of significantly differentiating between different magnitude levels. mFreEn had a higher stability of entropy values compared to mvSampEn for different *m* as well as for different *p*-value levels.

#### 3.1.3. Analysis of Frequency Detection Capability Based on MIX Model

**Figure 5:**
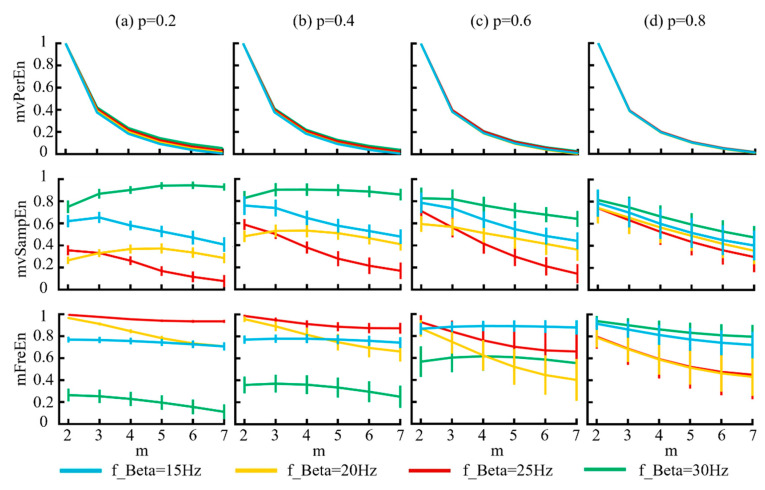
The mean and SD of normalized entropy values were calculated for signals generated from the MIX model, which demonstrated varying frequencies. The results corresponding to the four distinct *p* values are displayed in panels (**a**–**d**).

By controlling the frequency of the beta band (15 Hz, 20 Hz, 25 Hz, and 30 Hz), using the MIX model we generated four different combinations of signals. The model parameters were delineated as follows: the probability parameter *p* was set as [0.2, 0.4, 0.6, 0.8], the sampling rate *f_s_* = 500 Hz, and *t* = 3 s. The simulated signals were filtered into theta (4–8 Hz), alpha (8–13 Hz), and beta (13–30 Hz) frequency bands. Figure 5 illustrates the variation in entropy across different embedding dimensions.

Figure 5 demonstrates that for the mvPerEn algorithm, the entropy values of the four signals exhibited a gradual decrease as the embedding dimension increased. In contrast, the entropy variations for mvSampEn and mFreEn were irregular. Some entropy values showed a consistent decline, whereas others initially increased before decreasing. Considering the magnitude of the entropy values, mFreEn showed a less pronounced decrease with increasing embedding dimensions, indicating a reduced sensitivity to the embedding dimension. The classification performance of both mvSampEn and mFreEn for the four signals demonstrated similarity, showing a significant improvement over that of mvPerEn.

#### 3.1.4. Noise Robustness Analysis

**Figure 6:**
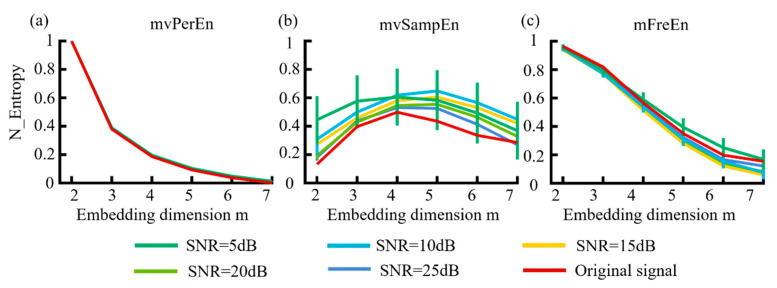
The mean and SD of normalized entropy values were obtained using (**a**) mvPerEn, (**b**) mvSampEn, and (**c**) mFreEn at different levels of noise intensity across datasets consisting of 1500 data points each.

WGN at levels of 5 dB, 10 dB, 15 dB, 20 dB, and 25 dB was added to the MIX model data to generate simulated signals with various SNRs. The probability parameter was set to *p* = 0.2. The sampling rate *fs* = 500 Hz, *t* = 3 s. Each dataset was generated 20 times. The simulated signals were filtered into theta (4–8 Hz), alpha (8–13 Hz), and beta (13–30 Hz) frequency bands. The means and SDs of the normalized entropy values were computed for each algorithm and are presented in Figure 6.

Figure 6 indicates that the mFreEn algorithm demonstrated a good anti-noise performance at embedding dimensions m = 2, 3, and 4. The anti-noise performance slightly weakened at m = 5, 6, and 7, which aided in the selection of embedding dimensions. In contrast, the mvSampEn algorithm was greatly affected by noise, showing significant deviations in the signal across all embedding dimensions. The mvPerEn entropy algorithm maintained robust noise resistance at all noise levels. Additionally, as the embedding dimension m increased, the entropy values of the mvSampEn algorithm initially increased and then decreased, with unstable relationships between the curves. Meanwhile, the entropy values of mvPerEn and mFreEn showed a decreasing trend and were more stable.

#### 3.1.5. Effect of Data Length

**Figure 7:**
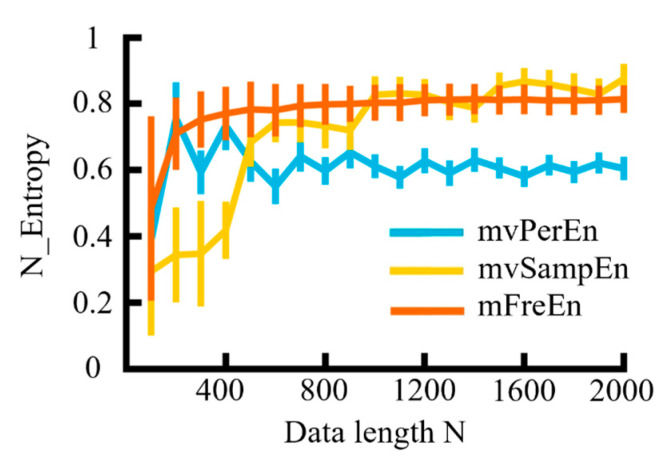
The mean and SD of entropy values were calculated for data lengths ranging from 100 to 2000, in increments of 100.

The MIX model was utilized for simulation analysis to examine the effects of data length on three distinct entropy algorithms. The sampling rate *f_s_* = 500 Hz, *t* = 3 s, and each generation was repeated 20 times. The simulated signals were filtered into theta (4–8 Hz), alpha (8–13 Hz), and beta (13–30 Hz) frequency bands. Based on the previous simulation results, the anti-noise performance of *m* is weakened when it exceeds 4 and if the value is too small, the sequence contains too few states. Therefore, m was proposed to be set to 4. The data length N varied from 100 to 2000 in increments of 100 and the entropy values of several algorithms were normalized. The means and SD of the normalized entropy values obtained were calculated for each of the three algorithms and are presented in Figure 7.

As observed in Figure 7, with the continuous increase in data length (N), the values of the three entropy algorithms demonstrated an upward trend and gradually stabilized. The entropy values of the mvPerEn algorithm stabilized at N = 700, and those of the mvSampEn algorithm at N = 1000, while the mFreEn algorithm reached stability at N = 400. In comparison to the mvPerEn and mvSampEn algorithms, the mFreEn algorithm could yield stable entropy values even with relatively limited data points. Overall, this indicated that the data length requirement for applying the mFreEn algorithm was much lower than that for mvPerEn and mvSampEn.

### 3.2. Application of Algorithms Based on Real EEG Data

#### 3.2.1. The Diagnosis of Brain Diseases

**Figure 8:**
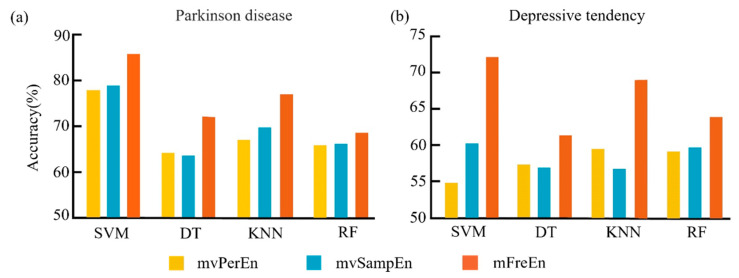
The mean classification accuracy was achieved with the optimal parameters for the SVM, DT, KNN, and RF classifiers across the three entropy algorithms. The results for the two datasets are depicted in panels (**a**,**b**).

To assess the effectiveness of entropy algorithms in distinguishing between PD patients and healthy controls, as well as between individuals with low and high tendencies for depression, we divided the preprocessed dataset into three frequency bands: alpha, beta, and theta. Subsequently, we segmented the frequency-divided data into non-overlapping intervals, with each interval containing 200 time points. Entropy values for each segment were computed using three different entropy algorithms, with the threshold parameter set to *r* = 0.15 times the standard deviation and the embedding dimension set to *m* = 4. For each dataset, we randomly selected 80 percent of the subjects as the training set, and the remaining 20 percent as the test set. The input feature was represented as a *p* × *n* vector, where *p* denotes the number of channels and *n* represents the total number of intervals. Subsequently, we applied the Support Vector Machine (SVM), Decision Tree (DT), K-Nearest Neighbors (KNN), and Random Forest (RF) classifiers to compute the classification accuracy. In order to eliminate the influence of randomness, the dataset was repeatedly partitioned and the model was trained ten times. The optimal average classification accuracy, achieved after parameter optimization, is depicted in Figure 8.

Figure 8a clearly shows that for the Parkinson’s disease dataset, the mFreEn algorithm exhibited a superior classification accuracy in all four classifiers when compared to mvPerEn and mvSampEn. The mvSampEn algorithm surpassed mvPerEn in classification accuracy within the SVM, KNN, and RF models. However, it fell behind mvPerEn with the DT classifier. Figure 8b indicates that for the depressive tendency dataset, the mFreEn algorithm significantly surpassed both mvPerEn and mvSampEn in classification accuracy across all four classifiers. While the mvSampEn algorithm yielded a higher accuracy than mvPerEn in the SVM and RF classifiers, it was outperformed in the DT and KNN classifiers.

#### 3.2.2. The Classification of Different Tasks

For task-related data, we continued to divide the data from each channel into three frequency bands: alpha, beta, and theta. We segmented the frequency-divided data using a sliding window, with each segment being 200 data points in length and a step size increment of 1. Subsequently, entropy values for each time segment were determined using three distinct entropy algorithms, setting the threshold parameter at *r* = 0.15 times the standard deviation and the embedding dimension at *m* = 4. To evaluate the capability of the three algorithms in distinguishing brain signals under various stimuli, the entropy values from different subjects, trials, and multiple channels within the same brain area were averaged, preserving only spatial frequency and time as dimensions. A baseline range was established from −600 to −200 ms, from which the baseline entropy values were subtracted at all respective time points, resulting in a line graph depicting the entropy fluctuations over time relative to the baseline.

**Figure 9:**
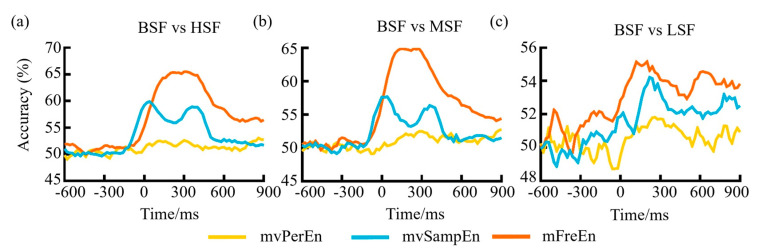
The classification accuracy variation over time for (**a**) BSF vs HSF, (**b**) BSF vs MSF, and (**c**) BSF vs LSF.

To evaluate the classification capabilities of the three entropy algorithms when applied to task-state data and test to which degree similar neural patterns occur at different time points of object-scene integration, a multivariate pattern analysis (MVPA) was implemented on the epoched EEG data. Regarding the three comparison scenarios: BSF versus LSF, BSF versus MSF, and BSF versus HSF, an SVM implemented in scikit-learn was trained to classify trials as being consistent or inconsistent based on brain activity. A three-fold stratified cross-validation procedure was used: each participant’s epochs were split into three equal sized folds. The fluctuation in the classification accuracy over time can be observed in Figure 9. As can be seen from Figure 9, before reaching −100 ms, the classification accuracy of the features gleaned from all three methodologies hovered consistently around the 50% threshold. After −100 ms, it began to rise, possibly due to the inclusion of data post-stimulation in the input feature matrix. Following the stimulation, the classification accuracy for mFreEn was higher than mvSampEn, and mvSampEn was higher than mvPerEn. This suggests that the mFreEn algorithm possesses superior discriminative ability for EEG signals obtained under various stimulations.

## 4. Discussion

In contrast to earlier studies that simply adapted multivariate entropy algorithms for assessing multi-channel signal complexity [48,49], this study proposed an innovative multivariate entropy algorithm, specifically designed to intricately analyze the time–frequency complexity associated with various frequency bands, termed mFreEn.

The new algorithm calculates complexity by measuring the permutation patterns of amplitude variations between frequency bands. To substantiate the efficacy of the novel algorithm, three distinct experimental facets were explored to assess the performance of the newly developed algorithm. In comparison to mvPerEn and mvSampEn, mFreEn demonstrated a superior performance in distinguishing signals of white Gaussian noise and 1/f noise. Upon analyzing the effects of amplitude, frequency, and data length on time series synthesized with the MIX model, we found that mFreEn could better distinguish signals of different amplitudes and frequency bands and had a reduced dependency on parameters (*m*, data length) compared to mvPerEn and mvSampEn. After conducting noise resistance experiments with simulated data, mvPerEn and mFreEn demonstrated robust noise resistance. Nonetheless, mvPerEn was concurrently observed to possess a lower discriminative capacity for sinusoidal signals across various frequency bands and amplitudes. Based on the cumulative findings of the simulation analysis, the mFreEn algorithm exhibits a good overall performance. Finally, this study incorporated three unique datasets of authentic EEG recordings, encompassing data from both resting and active task states. The findings revealed that mFreEn exhibited a superior performance in classification efficacy. Consequently, through a systematic analysis of the impacts of parameters, the robustness to noise, and the sensitivity using simulated and authentic EEG datasets, the overall performance of the mFreEn algorithm surpassed that of mvPerEn and mvSampEn.

(1) Parameter settings for the mFreEn algorithm

The settings of the different parameters of the algorithm are crucial in performing the analysis. For the three multivariate entropy algorithms used in this study, all of them involve the setting of the embedding dimension and the time delay. According to the type of embedding method, state space reconstruction is jointly controlled by the embedding dimension and the time delay. In most of the studies, uniform embedding was used [50,51]. Uniform embedding essentially sets the same time delay and embedding dimension for all channels. Although uniform embedding is a practical scheme widely used in the modeling of chaotic systems, there is evidence that nonuniform embedding may be more appropriate for multivariate time series [52,53,54].

Currently, there is an increasing interest in how to optimize the dimension of the joint embedding space. Faes et al. proposed the use of a conditional entropy criterion and sequential forward selection strategy for nonuniform multivariate embedding in one study [52]. In another study [54], they introduced a nonuniform approach by employing a step-by-step composition of embedding vectors to reduce conditional entropy. In addition, Wang et al. employed a straightforward and intuitive approach by generating a unique probability distribution for each channel in order to construct the phase space [55]. Furthermore, in the study by Porta et al., the optimization of the joint embedding space dimension was performed based on the Akaike figure of merit [56]. It has been shown that multivariate state space reconstruction using nonuniform embedding works better than uniform embedding. Thus, these studies demonstrate that nonuniform embedding can effectively reconstruct multivariate state spaces and potentially yield superior results compared to uniform embedding strategies.

For the mvSampEn and mvPerEn algorithms used in this study, there have also been some studies devoted to how to optimize the multivariate embedding space [57,58]. As we demonstrated in Figure 1, mvSampEn and mvPerEn are computed by combining the information from each channel in a measure of complexity. For both algorithms, it is feasible to perform the construction of nonuniform spaces by constructing the phase space with different structures to generate a unique probability distribution for each channel. For example, Xiao et al. assigned a different embedding dimension to every data channel when computing mvSampEn [57]. In the study by Li et al., they used probability theory to discuss all the strategies and proved that the m → m + p (p is the number of channels) is the best strategy to increase the embedding dimension vector [58]. However, for our proposed mFreEn algorithm, which needs to unite multiple band information for time domain and inter-band analysis at the same time, uniform embedding is more appropriate, and how to carry out nonuniform embedding in this case will be one of the research content points we consider in the future research, i.e., proposing variational embedding in mFreEn.

Therefore, in this study, we proposed the mFreEn algorithm and adopted uniform embedding to construct the multivariate embedding space. In order to be consistent, in both mvSampEn and mvPerEn, we also chose uniform embedding and set parameters that were consistent with mFreEn, in order to compare the algorithms intuitively. Studies have reported that the effect of time delay on entropy computation is very small, and it has generally been set to 1 for all experiments, so that the multivariate embedding space is fully defined by the change in embedding dimension. Therefore, in simulation experiments, we explored the performance of the algorithm and the embedding dimensions were set from 2 to 7.

From the results, we found that mFreEn was able to distinguish different combinations of signals compared to mvSampEn and mvPerEn. In addition, it was also found that the entropy value and entropy reliability appeared to decrease as the embedding dimension increased. This is consistent with what previous studies have reported [55,59]. Porta et al. provided a comparison and stated that model-free approaches are less efficient when applied to nonlinear systems under a high embedding dimension; these also exhibit lower reliability [59]. With a limited amount of data, it is inevitable that the reliability of complexity decreases gradually with the increase in the embedding dimension. Generally speaking, if *m* is too small, the reconstructed vectors will contain too few templets for matching, which will result in that the algorithm loses its validity and cannot accurately detect the dynamic change in time series. However, if *m* is too large, the reconstruction of the phase space will homogenize the time series, which is time-consuming and does not reflect the small changes in the time series, leading to a decrease in the entropy value and entropy reliability. The ideal embedding dimension may differ across disorders and even among individual patients [60,61,62]. Therefore, in practical application, the appropriate embedding dimensions need to be determined based on the research.

(2) The heightened sensitivity of mFreEn

The heightened sensitivity of mFreEn was evident in its capacity to discern subtle signal variations, whether among simulated signals, diverse pathological states, or within an individual subjected to a variety of stimuli. The underlying reasons for these findings indicate that substantial information was encoded within the frequency bands [63,64]. The relative pattern shifts among these bands accurately reflect abnormalities in neural signals and variations in EEG recordings during disparate tasks. The method’s sensitivity amplifies its capacity to differentiate among various neurological conditions, thereby facilitating more precise diagnoses and tailored treatment planning. Moreover, mFreEn’s sensitivity permits an enhanced and nuanced analysis of EEG data, enabling advanced research into the complexities of cerebral function and disorders.

(3) The anti-noise performance of mFreEn

It should be noted that mvPerEn demonstrated superior noise immunity, with mFreEn exhibiting greater noise resilience compared to mvSampEn in the comparative noise immunity experiments. The superior anti-noise performance of PeEn and mvPerEn has been demonstrated, primarily because they consider only the permutation information of the signal [65]. mFreEn’s superior noise immunity compared to mvSampEn may stem from the minimal impact of noise on the patterns’ relative changes across various frequency bands. However, sensitivity experiments revealed that mvPerEn had difficulty in distinguishing among different types of signals such as white Gaussian noise and 1/f noise. Thus, mvPerEn demonstrated limitations in effectively analyzing frequency band information due to its focus on amplitude variations without considering their magnitude. In practical applications, the brainwave signals collected invariably contain noise. Both strong noise resistance and high sensitivity are required to extract higher-quality features from the time series.

Therefore, our experimental results indicated that mFreEn achieved an optimal comprehensive performance. The performance of the mFreEn algorithm has significantly improved because it measures relative amplitude changes across various frequency bands, thus providing a more nuanced extraction of inter-frequency band information. The introduction of mFreEn in EEG signal analysis could improve the understanding and diagnosis of neurological conditions. With its low dependency on parameters, strong resistance to noise, and high sensitivity, mFreEn could be recognized as a highly effective tool for clinical and research applications, which paves the way for the development of more precise and personalized treatments for mental disorders.

## 5. Limitations

In this study, the mFreEn algorithm was proposed and validated using simulated and real EEG data. Preliminary experimental results have demonstrated the better overall performance of mFreEn compared to mvSampEn and mvPerEn. However, the study still has several limitations. A limitation of this study is the parameter settings of the mFreEn algorithm and the setting of different time delays, as well as the consideration of nonuniform embedding techniques which are the direction of our further research. One potential limitation of the filtering procedure approach is that it may prevent the authors from accurately detecting cross-coupling interferences. How to overcome the potential limitations of the filtering procedure approach will also be a direction for our future research.

## 6. Conclusions

Multivariate entropy effectively quantifies the nonlinear information embedded within multiple time series. In this study, we introduced a novel multivariate entropy algorithm, mFreEn, designed to analyze signals across various frequency bands by designating them as the variables of interest. The mFreEn algorithm, capable of simultaneously extracting information from both the time domain and frequency domain, provides a powerful tool for advancing the exploration of the interrelationships among different frequency bands. Compared to traditional multivariate entropy algorithms, mFreEn has demonstrated elevated sensitivity, reduced parameter dependency, and robust noise resistance. Upon applying mFreEn to EEG signal analysis, we observed that mFreEn exhibited a good classification performance for different brain disorders and EEG activity induced by diverse task stimuli. mFreEn is expected to emerge as a powerful algorithm for the extraction of essential physiological data.

Future work can be considered from several aspects. Firstly, multi-scale mFreEn can be proposed on the basis of mFreEn to study the change in complexity in different scales. Secondly, the nonuniform embedding method can be considered when constructing the multivariate embedding space. Finally, the application of mFreEn to signals like electrocardiograms and nuclear magnetic resonance imaging is also a prospective application under consideration.

## Data Availability

Data will be made available on request.

## Supplementary Materials

The following supporting information can be downloaded at: https://www.mdpi.com/article/10.3390/e26090728/s1. Datasets and Preprocessing [35].
