# Supplementary material for: Multi-Frequency Entropy for Quantifying Complex Dynamics and Its Application on EEG Data
Source: Entropy (Basel). 2024 Aug 27;26(9):728. doi: 10.3390/e26090728 (PMC11431093; doi:10.3390/e26090728)
Supplement: Supplementary file 1 [file entropy-26-00728-s001.zip › entropy-3088216-supplementary.pdf]

entropy MDPI

Supplementary Materials. Datasets and Preprocessing.

1. Resting-State EEG

(1) Parkinson disease dataset and preprocessing

The EEG data used in this study were obtained from 14 Parkinson's disease (PD) patients and 14 healthy controls at the University of Iowa (UI; Iowa City, Iowa) (the data can be obtained from the website https://bit.ly/3pP1pts and accessed on 5 April 2023). Resting-state EEG recordings of these 28 subjects from Iowa were collected with their eyes open. EEG signals were acquired using sintered Ag/AgCl electrodes in the frequency range of 0.1–100 Hz and at a sampling rate of 500 Sa/s on a 64-channel Brain Vision system, with the online reference designated to channel Pz (Iowa) for baseline measurements. Therefore, Pz channel data were not available in the Iowa datasets. The EEG data from the remaining 62 channels were used. Artifacts from eye blinks were identified and removed using independent component analysis. Further details about the dataset can be found in Reference [35].

EEG preprocessing was conducted offline using the EEGLAB v2019.1 toolbox within MATLAB R2018b (MathWorks, Natick, MA, USA). The data underwent bandpass filtering within the 0.1 to 40 Hz frequency range. Channels FT9, TP9, TP10, PO3, and PO4 were excluded owing to suboptimal data quality. Consequently, a total of 59 channels were retained for analysis. Subsequently, independent component analysis (ICA) was employed to isolate artifacts within the EEG signal, utilizing the logistic infomax ICA algorithm via the runica function in EEGLAB. Components manifesting dominant artifacts, including eye movements, muscle contractions, and cardiac activity, were excised.

EEG preprocessing was performed offline using the EEGLAB v2019.1 toolbox within MATLAB R2018b (MathWorks, Natick, MA, USA). The data were bandpass filtered in the frequency range of 0.1 to 40 Hz. Channels FT9, TP9, TP10, PO3, and PO4 were excluded due to suboptimal data quality, resulting in a total of 59 channels being retained for further analysis. Independent component analysis (ICA) was then applied to remove artifacts from the EEG signal, using the logistic infomax ICA algorithm via the runica function in EEGLAB. Components showing dominant artifacts, such as eye movements, muscle contractions, and cardiac activity, were removed.

(2) Depressive tendency's dataset and preprocessing

EEG signals were recorded from twenty-eight healthy volunteers at the Shenzhen Institute of Advanced Technology, Chinese Academy of Sciences. The participants included 14 individuals without depressive tendencies and 14 individuals with depressive tendencies, as assessed by the Beck Depression Inventory Scale scores. The recordings took place in a quiet, dimly lit room. Participants self-reported as right-handed and had either normal vision or corrected-to-normal vision. None of the participants reported a history of neurological problems. Prior to testing, informed consent was obtained from all participants.

EEG data were recorded using Grael amplifiers connected to 32 electrodes following the international 10-10 electrode system. Participants were seated in a comfortable armchair and instructed to keep their eyes open, fixating on a white visual stimulus. The bilateral mastoid processes served as reference electrodes. Electrode impedance was kept below 5 kΩ, and the sampling frequency was set at 500 Sa/s. The GND electrode acted as the ground, while the REF electrode provided the reference. Eye movements were monitored using a horizontal bipolar electrode (HEOG) placed approximately 1 cm from the outer canthus of the left eye and a vertical bipolar electrode (VEOG) positioned about 1.5 cm below the left eye. EEG signals were recorded continuously for five minutes.

The EEG data were preprocessed offline using the EEGLAB v2019.1 toolbox in MATLAB R2018b (MathWorks, Natick, MA, USA). The data were bandpass filtered

within the 0.1 to 40 Hz frequency range. Independent component analysis (ICA) was then applied to identify and remove artifacts from the EEG signal. Channels HEOG, VEOG, M1, M2, Fp1, and Fp2 were excluded from the analysis. The logistic infomax ICA algorithm within the runica function of EEGLAB was used for this purpose. Components primarily associated with artifacts such as eye movements, muscle contractions, and cardiac activity were removed from further analysis.

2. Task-State EEG and Preprocessing

The task-state EEG datasets were obtained from Okayama University in Japan and included thirty-two healthy volunteers (mean age = 24.63 years, SD = 3.06; age range 21–32 years; comprising 24 males and 8 females). EEG data were recorded using BrainAmp MR plus amplifiers (Gilching, Germany) with 64 AgCl electrodes placed according to the international 10-10 electrode system and affixed to an electrode cap (Easy-cap, Herrsching Breitbrunn, Germany). The AFz electrode was used as the ground. Two reference electrodes were positioned on the left and right earlobes. Eye movements were tracked using a horizontal bipolar electrode (HEOG) located approximately 1 cm from the outer canthus of the left eye and a vertical bipolar electrode (VEOG) positioned about 1.5 cm below the left eye. The EEG signals were digitized at a sampling rate of 500 Sa/s. The primary experiment utilized a within-subject design with four conditions: basic spatial frequency (BSF), low spatial frequency (LSF), medium spatial frequency (MSF), and high spatial frequency (HSF).

The EEG data were preprocessed offline using the EEGLAB v2019.1 toolbox in MATLAB R2018b (MathWorks, Natick, MA, USA). The data were bandpass filtered within the 0.1 to 40 Hz frequency range. Independent component analysis (ICA) was then applied to identify and remove artifacts from the EEG signal. Channels HEOG, VEOG, M1, and M2 were excluded from the analysis. The logistic infomax ICA algorithm within the runica function of EEGLAB was used for this purpose. Components primarily associated with artifacts such as eye movements, muscle contractions, and cardiac activity were removed from further analysis.

In addition, for the task-state dataset, the cleaned EEG data were segmented from 800 ms before stimulus onset to 1200 ms after stimulus presentation. The reference was recalculated offline using the mean of all electrodes, excluding HEOG, VEOG, M1, and M2. Following re-referencing, trials underwent baseline correction using the prestimulus interval (−200 to 0 ms) as the reference period.
